# Supplementary material for: Clinical and pharmacokinetic/dynamic outcomes of prolonged infusions of beta-lactam antimicrobials: An overview of systematic reviews
Source: PLoS One. 2021 Jan 22;16(1):e0244966. doi: 10.1371/journal.pone.0244966 (PMC7822342; doi:10.1371/journal.pone.0244966)
Supplement: S1 Table — AMSTAR criterion: 1 –Did the research question and inclusion criteria for the review include the components of PICO?; 2 –Did the report of the review contain an explicit statement that the review methods were established prior to the conduct of the review and did the report justify any significant deviations from the protocol?; 3 –Did the review authors explain their selection of the study designs for inclusion in the review?; 4 –Did the review authors use a comprehensive literature search strategy?; 5 –Did the review authors perform study selection in duplicate?; 6 –Did the review authors perform data extraction in duplication?; 7 –Did the review authors provide a list of excluded studies and justify the exclusions?; 8 –Did the review authors describe the included studies in adequate detail?; 9 –Did the review authors use a satisfactory technique for assessing ROB in individual studies that were included in the review? 9a –RCT, 9b –NRSI; 10 –Did the review authors report on the sources of funding for the studies included in the review?; 11 –If meta-analysis was performed did the review authors use appropriate methods for statistical combination of results? 11a –RCT; 11b –NRSA; 12 –If meta-analysis was performed, did the review authors assess the potential impact of ROB in individual studies on the results of the meta-analysis or other evidence synthesis?; 13 –Did the review authors account for ROB in individual studies when interpreting/discussing the results of the review?; 14 –Did the review authors provide a satisfactory explanation for, and discussion of, any heterogeneity observed in the results of the review?; 15 –If they performed quantitative synthesis did the review authors carry out an adequate investigation of publication bias (Small study bias) and discuss its likely impact on the results of the review?; 16 –Did the review authors report any potential sources of conflict of interest, including any funding they received for conducting the review? Abbrevi [file pone.0244966.s001.docx]

**S1 Table.** **AMSTAR results of included reviews**

|  | 1 | 2 | 3 | 4 | 5 | 6 | 7 | 8 | 9a | 9b | 10 | 11a | 11b | 12 | 13 | 14 | 15 | 16 | Overall |
| --- | --- | --- | --- | --- | --- | --- | --- | --- | --- | --- | --- | --- | --- | --- | --- | --- | --- | --- | --- |
| Rhodes 2018 | Y | N | N | PY | N | Y | N | PY | Y | N | N | N | N | N | N | N | Y | N | CL |
| Vardakas 2018 | Y | Y | N | PY | Y | Y | N | Y | Y | NA | N | Y | NA | N | N | Y | Y | Y | L |
| Yu 2018 | Y | N | N | PY | Y | Y | N | PY | Y | Y | N | Y | N | Y | Y | N | Y | Y | L |
| Lee 2017 | Y | Y | N | N | Y | Y | N | N | N | N | Y | Y | NA | N | N | N | Y | Y | CL |
| Lal 2016 | Y | N | N | PY | Y | Y | N | PY | PY | Y | N | Y | NA | N | N | Y | N | Y | L |
| Roberts 2016 | Y | N | Y | N | N | Y | N | PY | Y | NA | N | Y | NA | Y | Y | Y | Y | Y | L |
| Yang 2016 | Y | N | N | PY | Y | Y | PY | PY | Y | Y | N | Y | N | Y | Y | N | Y | Y | L |
| Burgess 2015 | Y | N | N | PY | N | N | N | PY | N | N | N | NA | NA | NA | N | N | NA | Y | CL |
| Yang 2015 | Y | N | N | PY | N | Y | N | PY | Y | Y | N | Y | N | N | Y | Y | Y | Y | L |
| Lux 2014 | Y | Y | Y | Y | Y | Y | PY | PY | Y | Y | Y | NA | NA | NA | Y | Y | NA | Y | M |
| Teo 2014 | Y | N | N | PY | Y | N | PY | PY | Y | Y | N | Y | N | N | N | Y | Y | Y | L |
| Yusuf 2014^a^ | Y | Y | N | N | Y | N | N | PY | Y | Y | N | NA | NA | NA | N | N | NA | Y | CL |
| Chant 2013 | Y | N | N | PY | Y | Y | PY | PY | PY | N | N | Y | N | N | N | N | Y | Y | CL |
| Falagas 2013 | Y | N | N | PY | N | N | PY | PY | N | N | N | N | N | N | N | N | N | Y | CL |
| Korbila 2013 | Y | N | N | N | N | N | N | Y | N | N | N | N | NA | N | N | N | N | Y | CL |
| Hassan  2013 | N | N | N | N | Y | N | N | PY | N | NA | Y | Y | NA | N | N | Y | Y | Y | CL |
| Garcia 2012 | Y | N | N | N | N | Y | N | N | N | N | N | NA | NA | NA | N | N | NA | Y | CL |
| Mah 2012 | Y | N | N | PY | N | N | N | PY | N | N | N | NA | NA | NA | N | Y | NA | N | CL |
| Tamma 2011 | Y | N | Y | PY | Y | Y | PY | PY | Y | NA | Y | Y | NA | Y | Y | N | Y | Y | M |
| Roberts 2009 | Y | N | N | PY | Y | Y | PY | PY | N | NA | Y | N | NA | N | N | Y | Y | Y | CL |
| Roberts 2007 | Y | Y | N | PY | N | N | N | N | N | N | N | NA | NA | NA | N | Y | NA | N | CL |

AMSTAR criterion: 1 – Did the research question and inclusion criteria for the review include the components of PICO?; 2 – Did the report of the review contain an explicit statement that the review methods were established prior to the conduct of the review and did the report justify any significant deviations from the protocol?; 3 – Did the review authors explain their selection of the study designs for inclusion in the review?; 4 – Did the review authors use a comprehensive literature search strategy?; 5 – Did the review authors perform study selection in duplicate?; 6 – Did the review authors perform data extraction in duplication?; 7 – Did the review authors provide a list of excluded studies and justify the exclusions?; 8 – Did the review authors describe the included studies in adequate detail?; 9 – Did the review authors use a satisfactory technique for assessing ROB in individual studies that were included in the review? 9a – RCT, 9b – NRSI; 10 – Did the review authors report on the sources of funding for the studies included in the review?; 11 – If meta-analysis was performed did the review authors use appropriate methods for statistical combination of results? 11a – RCT; 11b – NRSA; 12 – If meta-analysis was performed, did the review authors assess the potential impact of ROB in individual studies on the results of the meta-analysis or other evidence synthesis?; 13 – Did the review authors account for ROB in individual studies when interpreting/discussing the results of the review?; 14 – Did the review authors provide a satisfactory explanation for, and discussion of, any heterogeneity observed in the results of the review?; 15 – If they performed quantitative synthesis did the review authors carry out an adequate investigation of publication bias (Small study bias) and discuss its likely impact on the results of the review?; 16 – Did the review authors report any potential sources of conflict of interest, including any funding they received for conducting the review?

Abbreviations: Y – Yes; PY – Partial Yes; N – No; NA – Not Applicable; CL – critically low quality; L – low quality; M – moderate quality.
