# Supplementary material for: Clinical and pharmacokinetic/dynamic outcomes of prolonged infusions of beta-lactam antimicrobials: An overview of systematic reviews
Source: PLoS One. 2021 Jan 22;16(1):e0244966. doi: 10.1371/journal.pone.0244966 (PMC7822342; doi:10.1371/journal.pone.0244966)
Supplement: S2 Table — NOS—Newcastle Ottawa Scale; ROB—Risk of bias. (DOCX) [file pone.0244966.s002.docx]

**S2 Table.** **Risk of bias and/or quality assessments of primary studies as reported in included reviews**

| **Primary Study** | **# reviews in which primary study is included** | **# of reviews that assess ROB or quality of primary study** | **# of distinct scales or validated tools used for ROB/quality assessment** | **Description of ROB/quality assessment** |
| --- | --- | --- | --- | --- |
| Abdul-Aziz et al. 2016 a | 5(20, 22, 26, 29, 50) | 4(20, 22, 29, 50) | NOS  Cochrane Handbook  Unspecified | Varied ROB conclusions.  High ROB in one review.(50) |
| Abdul-Aziz et al. 2016 b | 1(29) | 1(29) | Jadad | High quality based on one review |
| Angus et al. 2000 | 6(21, 23, 24, 26, 47, 51) | 2(21, 24) | Jadad  Cochrane Handbook | High risk of bias in two reviews (21, 24) |
| Arnold et al. 2013 | 2(19, 45) | 1(45) | Tool not specified | Overall quality assessment not provided |
| Bao et al. 2016 | 2(22, 26) | 1(22) | Cochrane ROB tool | Likely high ROB in one review. Only aggregate data of all include studies reported. (26)  High risk of bias with regards to blinding identified in one review (22) |
| Bauer et al. 2013 | 1(19) | 0 | None | No ROB assessment performed at review level |
| Benko et al. 1996 | 1(51) | 0 | None | No ROB assessment performed at review level |
| Bodey et al. 1979 | 3(21, 24, 27) |  | Jadad  Cochrane Handbook | Low quality in one review (21)  Likely high ROB in one review. Individual criteria reported although overall ROB not described (24) |
| Brunetti et al. 2015 | 1(27) | 1(27) | NOS | High quality (27) |
| Buck et al. 2005 | 10(21, 23-28, 33, 46, 52) | 5(21, 24, 27, 28, 46) | Jadad  Cochrane Handbook  NOS | Variable quality and ROB conclusions.  High ROB in one review(21)  Likely high ROB in one review (24)  High quality in two reviews using NOS (27, 28)  One review did not report ROB assessment results (46) |

NOS – Newcastle Ottawa Scale; ROB – Risk of bias
