# Supplementary material for: Clinical and pharmacokinetic/dynamic outcomes of prolonged infusions of beta-lactam antimicrobials: An overview of systematic reviews
Source: PLoS One. 2021 Jan 22;16(1):e0244966. doi: 10.1371/journal.pone.0244966 (PMC7822342; doi:10.1371/journal.pone.0244966)
Supplement: S3 Table — a Subsequent sensitivity analyses conducted to separate randomized and non-randomized trials, PI—prolonged infusion, II—intermittent infusion, AMSTAR-2 –assessing the methodologic quality of systematic reviews, ROBIS—risk of bias tool for systematic reviews. (DOCX) [file pone.0244966.s003.docx]

**S3 Table.** **Characteristics of reviews reporting mortality**

| Review | Population | Intervention | Comparator | Drug | Meta-analysis | Combined randomized and non-randomized data? | Mortality benefit identified? | AMSTAR-2 | ROBIS |
| --- | --- | --- | --- | --- | --- | --- | --- | --- | --- |
| Rhodes 2018 | Acutely/critically ill hospitalized | PI | II | Piperacillin/Tazobactam | Yes | Yes | Yes | Critically low | Low |
| Vardakas 2018 | Adult patients with sepsis | PI | II | Anti-pseudomonal beta-lactam | Yes | No | Yes | Low | Low |
| Yu 2018 | Severe infections | PI | II | Meropenem | Yes | Yes | Yes | Low | Low |
| Lee 2017 | Critically ill patients with respiratory infections | CI | II | Beta-lactams | Yes | No | No | Critically low | High |
| Lal 2016 | Nosocomial pneumonia | PI | II | Beta-lactams | Yes | Yes^a^ | No | Low | Low |
| Roberts 2016 | Severe sepsis of septic shock | CI | II | Beta-lactams | Yes | No | Yes | Low | Low |
| Yang 2016 | Unspecified population | PI | II | Piperacillin/Tazobactam | Yes | Yes^a^ | Yes | Low | Low |
| Burgess 2015 | Unspecified population | PI | II | Cefepime | No | - | - | Critically low | High |
| Yang 2015 | Unspecified population | PI | II | Piperacillin/Tazobactam | Yes | Yes | Yes | Low | Low |
| Lux 2014 | Hospital acquired pneumonia | PI | II | Beta-lactams | No | - | - | Moderate | Low |
| Teo 2014 | Acute infections in hospitalized adults | PI | II | Beta-lactams | Yes | Yes^a^ | Yes | Low | High |
| Yusuf 2014^a^ | Critically ill patients | PI | II | Piperacillin/tazobactam | No | - | - | Critically low | High |
| Chant 2013 | Critically ill patients | PI | II | Time dependent anti-microbials | Yes | Yes^a^ | Yes | Critically low | High |
| Falagas 2013 | Unspecified population | PI | II | Carbapenems, piperacillin/tazobactam | Yes | Yes | Yes | Critically low | High |
| Korbila 2013 | Unspecified population | PI | II | Cephalosporins (3^rd^, 4^th^, 5^th^ generation) | Yes | No | No | Critically low | High |
| Garcia 2012 | Patients with acute infections susceptible to piperacillin/tazobactam | PI | II | Piperacillin/tazobactam | No | - | - | Critically low | High |
| Mah 2012 | Adults requiring piperacillin/tazobactam | PI | II | Piperacillin/tazobactam | No | - | - | Critically low | High |
| Tamma 2011 | Unspecified population | PI | II | Beta-lactams | Yes | No | No | Moderate | Low |
| Roberts 2009 | Hospitalized adults with acute infection | PI | II | Beta-lactams | Yes | No | No | Critically low | High |
| Roberts 2007 | Serious infection | CI | II | Beta-lactams | No | - | - | Critically low | High |

^a^ Subsequent sensitivity analyses conducted to separate randomized and non-randomized trials, PI – prolonged infusion, II – intermittent infusion, AMSTAR-2 – assessing the methodologic quality of systematic reviews, ROBIS – risk of bias tool for systematic reviews
