# Supplementary material for: Clinical and pharmacokinetic/dynamic outcomes of prolonged infusions of beta-lactam antimicrobials: An overview of systematic reviews
Source: PLoS One. 2021 Jan 22;16(1):e0244966. doi: 10.1371/journal.pone.0244966 (PMC7822342; doi:10.1371/journal.pone.0244966)
Supplement: S5 Table — PI—prolonged infusion, II—intermittent infusion, AMSTAR-2 –assessing the methodologic quality of systematic reviews, ROBIS—risk of bias tool for systematic reviews. (DOCX) [file pone.0244966.s005.docx]

**S5 Table.** **Characteristics of reviews reporting microbiologic cure**

| Review | Population | Intervention | Comparator | Drug | Meta-analysis | Combined randomized and non-randomized data? | Microbiologic cure benefit identified? | AMSTAR-2 | ROBIS |
| --- | --- | --- | --- | --- | --- | --- | --- | --- | --- |
| Rhodes 2018 | Acutely/critically ill hospitalized | PI | II | Piperacillin/Tazobactam | Yes | Yes | No | Critically low | Low |
| Yu 2018 | Severe infections | PI | II | Meropenem | Yes | Yes | Yes | Low | Low |
| Lal 2016 | Nosocomial pneumonia | PI | II | Beta-lactams | Yes | Yes | No | Low | Low |
| Yang 2015 | Unspecified population | PI | II | Piperacillin/Tazobactam | Yes | Yes | No | Low | Low |

PI – prolonged infusion, II – intermittent infusion, AMSTAR-2 – assessing the methodologic quality of systematic reviews, ROBIS – risk of bias tool for systematic reviews
