# Supplementary material for: Clinical and pharmacokinetic/dynamic outcomes of prolonged infusions of beta-lactam antimicrobials: An overview of systematic reviews
Source: PLoS One. 2021 Jan 22;16(1):e0244966. doi: 10.1371/journal.pone.0244966 (PMC7822342; doi:10.1371/journal.pone.0244966)
Supplement: S6 Table — a Subsequent sensitivity analyses conducted to separate randomized and non-randomized trials, CI—Continuous infusion, PI—prolonged infusion, II—intermittent infusion, AMSTAR-2 –assessing the methodologic quality of systematic reviews, ROBIS—risk of bias tool for systematic reviews. (DOCX) [file pone.0244966.s006.docx]

**S6 Table.** **Characteristics of reviews reporting length of stay**

| Review | Population | Intervention | Comparator | Drug | Meta-analysis | Combined randomized and non-randomized data? | Length of stay benefit identified? | AMSTAR-2 | ROBIS |
| --- | --- | --- | --- | --- | --- | --- | --- | --- | --- |
| Rhodes 2018 | Acutely/critically ill hospitalized | PI | II | Piperacillin/Tazobactam | Yes | Yes | No | Critically low | Low |
| Yu 2018 | Severe infections | PI | II | Meropenem | No | - | - | Low | Low |
| Yang 2016 | Unspecified population | PI | II | Piperacillin/Tazobactam | No | - | - | Low | Low |
| Burgess 2015 | Unspecified population | PI | II | Cefepime | No | - | - | Critically low | High |
| Yusuf 2014^a^ | Critically ill patients | PI | II | Piperacillin/tazobactam | No | - | - | Critically low | High |
| Chant 2013 | Critically ill patients | PI | II | Time dependent anti-microbials | Yes | Yes^a^ | No | Critically low | High |
| Mah 2012 | Adults requiring piperacillin/tazobactam | PI | II | Piperacillin/tazobactam | No | - | - | Critically low | High |
| Roberts 2007 | Serious infection | CI | II | Beta-lactams | No | - | - | Critically low | High |

^a^ Subsequent sensitivity analyses conducted to separate randomized and non-randomized trials, CI – Continuous infusion, PI – prolonged infusion, II – intermittent infusion, AMSTAR-2 – assessing the methodologic quality of systematic reviews, ROBIS – risk of bias tool for systematic reviews
