# Supplementary material for: Clinical and pharmacokinetic/dynamic outcomes of prolonged infusions of beta-lactam antimicrobials: An overview of systematic reviews
Source: PLoS One. 2021 Jan 22;16(1):e0244966. doi: 10.1371/journal.pone.0244966 (PMC7822342; doi:10.1371/journal.pone.0244966)
Supplement: S7 Table — CI—Continuous infusion, PI—prolonged infusion, II—intermittent infusion, AMSTAR-2 –assessing the methodologic quality of systematic reviews, ROBIS—risk of bias tool for systematic reviews. (DOCX) [file pone.0244966.s007.docx]

**S7 Table.** **Characteristics of reviews reporting adverse events**

| Review | Population | Intervention | Comparator | Drug | Meta-analysis | Combined randomized and non-randomized data? | Adverse event difference identified? | AMSTAR-2 | ROBIS |
| --- | --- | --- | --- | --- | --- | --- | --- | --- | --- |
| Vardakas 2018 | Adult patients with sepsis | PI | II | Anti-pseudomonal beta-lactam | Yes | No | No | Low | Low |
| Yu 2018 | Severe infections | PI | II | Meropenem | No | - | - | Low | Low |
| Lal 2016 | Nosocomial pneumonia | PI | II | Beta-lactams | No | - | - | Low | Low |
| Burgess 2015 | Unspecified population | PI | II | Cefepime | No | - | - | Critically low | High |
| Yang 2015 | Unspecified population | PI | II | Piperacillin/Tazobactam | No | - | - | Low | Low |
| Lux 2014 | Hospital acquired pneumonia | PI | II | Beta-lactams | No | - | - | Moderate | Low |
| Teo 2014 | Acute infections in hospitalized adults | PI | II | Beta-lactams | No | - | - | Low | High |
| Falagas 2013 | Unspecified population | PI | II | Carbapenems, piperacillin/tazobactam | No | - | - | Critically low | High |
| Korbila 2013 | Unspecified population | PI | II | Cephalosporins (3^rd^, 4^th^, 5^th^ generation) | No | - | - | Critically low | High |
| Garcia 2012 | Patients with acute infections susceptible to piperacillin/tazobactam | PI | II | Piperacillin/tazobactam | No | - | - | Critically low | High |
| Tamma 2011 | Unspecified population | PI | II | Beta-lactams | No | - | - | Moderate | Low |
| Roberts 2007 | Serious infection | CI | II | Beta-lactams | No | - | - | Critically low | High |

CI – Continuous infusion, PI – prolonged infusion, II – intermittent infusion, AMSTAR-2 – assessing the methodologic quality of systematic reviews, ROBIS – risk of bias tool for systematic reviews
