# Supplementary material for: Clinical and pharmacokinetic/dynamic outcomes of prolonged infusions of beta-lactam antimicrobials: An overview of systematic reviews
Source: PLoS One. 2021 Jan 22;16(1):e0244966. doi: 10.1371/journal.pone.0244966 (PMC7822342; doi:10.1371/journal.pone.0244966)
Supplement: S9 Table — PI—prolonged infusion, II—intermittent infusion, AMSTAR-2—assessing the methodologic quality of systematic reviews, ROBIS—risk of bias tool for systematic reviews. (DOCX) [file pone.0244966.s009.docx]

**S9 Table.** **Characteristics of reviews reporting emergence of resistance**

| Review | Population | Intervention | Comparator | Drug | Meta-analysis | Combined randomized and non-randomized data? | Emergence of resistance benefit identified? | AMSTAR-2 | ROBIS |
| --- | --- | --- | --- | --- | --- | --- | --- | --- | --- |
| Vardakas 2018 | Adult patients with sepsis | PI | II | Anti-pseudomonal beta-lactam | Yes | No | No | Low | Low |
| Lux 2014^a^ | Hospital acquired pneumonia | PI | II | Beta-lactams | No | - | - | Moderate | Low |
| Falagas 2013 | Unspecified population | PI | II | Carbapenems, piperacillin/tazobactam | No | - | - | Critically low | High |
| Korbila 2013 | Unspecified population | PI | II | Cephalosporins (3^rd^, 4^th^, 5^th^ generation) | No | - | - | Critically low | High |

PI – prolonged infusion, II – intermittent infusion, AMSTAR-2 – assessing the methodologic quality of systematic reviews, ROBIS – risk of bias tool for systematic reviews
