# Supplementary material for: Clinical and pharmacokinetic/dynamic outcomes of prolonged infusions of beta-lactam antimicrobials: An overview of systematic reviews
Source: PLoS One. 2021 Jan 22;16(1):e0244966. doi: 10.1371/journal.pone.0244966 (PMC7822342; doi:10.1371/journal.pone.0244966)
Supplement: S10 Table — PI—prolonged infusion, II—intermittent infusion, AMSTAR-2 –assessing the methodologic quality of systematic reviews, ROBIS—risk of bias tool for systematic reviews. (DOCX) [file pone.0244966.s010.docx]

**S10 Table.** **Characteristics of reviews reporting PK/PD outcomes**

| Review | Population | Intervention | Comparator | Drug | Meta-analysis | Combined randomized and non-randomized data? | PK/PD benefit identified? | AMSTAR-2 | ROBIS |
| --- | --- | --- | --- | --- | --- | --- | --- | --- | --- |
| Lee 2017 | Critically ill patients with respiratory infections | CI | II | Beta-lactams | No | No | Yes | Critically low | High |
| Burgess 2015^a^ | Unspecified population | PI | II | Cefepime | No | Yes | Yes | Critically low | Low |
| Lux 2014^a^ | Hospital acquired pneumonia | PI | II | Beta-lactams | No | No | No | Moderate | Low |
| Garcia 2012 | Patients with acute infections susceptible to piperacillin/tazobactam | PI | II | Piperacillin/tazobactam | No | No | No | Critically low | High |
| Mah 2012^a^ | Adults requiring piperacillin/tazobactam | PI | II | Piperacillin/tazobactam | No | Yes | No | Critically low | High |
| Roberts 2007^a^ | Serious infection | CI | II | Beta-lactams | No | No | No | Critically low | High |

PI – prolonged infusion, II – intermittent infusion, AMSTAR-2 – assessing the methodologic quality of systematic reviews, ROBIS – risk of bias tool for systematic reviews
