# Supplementary material for: Clinical and pharmacokinetic/dynamic outcomes of prolonged infusions of beta-lactam antimicrobials: An overview of systematic reviews
Source: PLoS One. 2021 Jan 22;16(1):e0244966. doi: 10.1371/journal.pone.0244966 (PMC7822342; doi:10.1371/journal.pone.0244966)
Supplement: S1 Fig — (DOCX) [file pone.0244966.s011.docx]

**S1 Fig. Search Strategy**

Ovid Multifile

Database: Embase Classic+Embase <1947 to 2018 December 07>, Ovid MEDLINE(R) ALL <1946 to December 06, 2018>, EBM Reviews - Cochrane Database of Systematic Reviews <2005 to December 5, 2018>, EBM Reviews - Database of Abstracts of Reviews of Effects <1st Quarter 2016>, EBM Reviews - Health Technology Assessment <4th Quarter 2016>

Search Strategy:

--------------------------------------------------------------------------------

1 Anti-Bacterial Agents/ (462942)

2 exp beta-Lactams/ (129374)

3 beta-Lactam?.tw,kf. (44420)

4 (aztreonam? or az-threonam? or azactam or azthreonam or urobactam).tw,kf. (7427)

5 carbapenem?.tw,kf. (24783)

6 (cefepime or axepim or BMY 28142 or BMY-28142 or cefepim or maxipime or quadrocef).tw,kf. (8123)

7 cephalosporin?.tw,kf. (47576)

8 (imipemide or imipenem? or MK-0787 or MK0787 or N-Formimidoylthienamycin).tw,kf. (23808)

9 (meropenem? or merrem or penem or ronem or SM 7338).tw,kf. (15973)

10 (monobactam? or mono bactam?).tw,kf. (1647)

11 (moxalactam? or mox alactam? or disodium latamoxef or festamoxin or lamoxactam or latamoxef or shiomarin).tw,kf. (3625)

12 penicillin?.tw,kf. (136870)

13 (piperacillin? or pipcil or pipracil or pipril or T-1220 or T1220).tw,kf. (16728)

14 (tazobactam? or taszobactam sodium or YTR 830 or YTR 830H).tw,kf. (11362)

15 thienamycin?.tw,kf. (770)

16 or/1-15 [BETA-LACTAM ABX] (707467)

17 Drug Administration Routes/ (12844)

18 Drug Administration Schedule/ (148272)

19 drug administration*.tw,kf. (119692)

20 exp Administration, Intravenous/ (521446)

21 infusion?.tw,kf. (570959)

22 intravenous*.tw,kf. (781524)

23 (bolus or boluses).tw,kf. (126566)

24 ((continuous* or daily or extend* or intermittent* or longer or "multiple-daily" or "once-daily" or optimal* or optimis* or optimiz* or prolong* or "short-course" or "short-term") adj3 (administer* or administration*)).tw,kf. (110698)

25 ((continuous* or daily or extend* or intermittent* or longer or "multiple-daily" or "once-daily" or optimal* or optimis* or optimiz* or prolong* or "short-course" or "short-term") adj3 (dosage* or dose or doses or dosing or regimen? or infus* or IV or "I.V.")).tw,kf. (329674)

26 (method? adj2 (adminster* or administration*)).tw,kf. (13264)

27 (route? adj2 (adminster* or administration*)).tw,kf. (37473)

28 Anti-Bacterial Agents/ad [administration & dosage] (48909)

29 exp beta-Lactams/ad [administration & dosage] (20798)

30 or/17-29 [DRUG ADMINISTRATION] (2134275)

31 16 and 30 [BETA-LACTAM ABX - ADMINISTRATION] (106999)

32 limit 31 to systematic reviews [Limit not valid in Embase,CDSR,DARE,CLHTA; records were retained] (37712)

33 meta analysis.pt. (94679)

34 exp meta-analysis as topic/ (56367)

35 (meta-analy* or metanaly* or metaanaly* or met analy* or integrative research or integrative review* or integrative overview* or research integration or research overview* or collaborative review*).tw,kf. (357686)

36 (systematic review* or systematic overview* or evidence-based review* or evidence-based overview* or (evidence adj3 (review* or overview*)) or meta-review* or meta-overview* or meta-synthes* or "review of reviews" or technology assessment* or HTA or HTAs).tw,kf. (449097)

37 exp Technology assessment, biomedical/ (24087)

38 (cochrane or health technology assessment or evidence report).jw. (49456)

39 (network adj (MA or MAs)).tw,kf. (18)

40 (NMA or NMAs).tw,kf. (4375)

41 indirect* compar*.tw,kf. (5394)

42 (indirect treatment* adj1 compar*).tw,kf. (662)

43 (mixed treatment* adj1 compar*).tw,kf. (1364)

44 (multiple treatment* adj1 compar*).tw,kf. (362)

45 (multi-treatment* adj1 compar*).tw,kf. (4)

46 simultaneous* compar*.tw,kf. (2191)

47 mixed comparison?.tw,kf. (99)

48 or/33-47 (746314)

49 31 and 48 (2122)

50 32 or 49 [REVIEWS] (37950)

51 exp Animals/ not (exp Animals/ and Humans/) (16925176)

52 50 not 51 [ANIMAL-ONLY REMOVED] (28194)

53 (comment or editorial or news or newspaper article).pt. (1863046)

54 (letter not (letter and randomized controlled trial)).pt. (2055263)

55 52 not (53 or 54) [OPINION PIECES REMOVED] (27522)

56 55 use medall [MEDLINE RECORDS] (2078)

57 antibiotic agent/ (309116)

58 exp beta lactam antibiotic/ (441546)

59 beta-Lactam?.tw,kw. (45112)

60 (aztreonam? or az-threonam? or azactam or azthreonam or urobactam).tw,kw. (7434)

61 carbapenem?.tw,kw. (25167)

62 (cefepime or axepim or BMY 28142 or BMY-28142 or cefepim or maxipime or quadrocef).tw,kw. (8152)

63 cephalosporin?.tw,kw. (48091)

64 (imipemide or imipenem? or MK-0787 or MK0787 or N-Formimidoylthienamycin).tw,kw. (23880)

65 (meropenem? or merrem or penem or ronem or SM 7338).tw,kw. (16057)

66 (monobactam? or mono bactam?).tw,kw. (1665)

67 (moxalactam? or mox alactam? or disodium latamoxef or festamoxin or lamoxactam or latamoxef or shiomarin).tw,kw. (3626)

68 penicillin?.tw,kw. (135302)

69 (piperacillin? or pipcil or pipracil or pipril or T-1220 or T1220).tw,kw. (16751)

70 (tazobactam? or taszobactam sodium or YTR 830 or YTR 830H).tw,kw. (11377)

71 thienamycin?.tw,kw. (772)

72 or/57-71 [BETA-LACTAM ABX] (785788)

73 drug administration/ (54598)

74 drug administration route/ (12844)

75 exp intravenous drug administration/ (383501)

76 drug administration*.tw,kw. (120549)

77 exp infusion/ (135615)

78 infusion?.tw,kw. (571094)

79 intravenous*.tw,kw. (783588)

80 bolus injection/ (10432)

81 (bolus or boluses).tw,kw. (126684)

82 ((continuous* or daily or extend* or intermittent* or longer or "multiple-daily" or "once-daily" or optimal* or optimis* or optimiz* or prolong* or "short-course" or "short-term") adj3 (administer* or administration*)).tw,kw. (110742)

83 ((continuous* or daily or extend* or intermittent* or longer or "multiple-daily" or "once-daily" or optimal* or optimis* or optimiz* or prolong* or "short-course" or "short-term") adj3 (dosage* or dose or doses or dosing or regimen? or infus* or IV or "I.V.")).tw,kw. (330126)

84 (method? adj2 (adminster* or administration*)).tw,kw. (13280)

85 (route? adj2 (adminster* or administration*)).tw,kw. (37773)

86 antibiotic agent/ad, iv, pa [Drug Administration, Intravenous Drug Administration, Parenteral Drug Administration] (3774)

87 exp beta lactam antibiotic/ad, iv, pa [Drug Administration, Intravenous Drug Administration, Parenteral Drug Administration] (10560)

88 or/73-87 [DRUG ADMINISTRATION] (2018116)

89 72 and 88 [BETA-LACTAM ABX - ADMINISTRATION] (99130)

90 meta-analysis/ (248723)

91 "systematic review"/ (187806)

92 "meta analysis (topic)"/ (39279)

93 (meta-analy* or metanaly* or metaanaly* or met analy* or integrative research or integrative review* or integrative overview* or research integration or research overview* or collaborative review*).tw,kw. (360493)

94 (systematic review* or systematic overview* or evidence-based review* or evidence-based overview* or (evidence adj3 (review* or overview*)) or meta-review* or meta-overview* or meta-synthes* or "review of reviews" or technology assessment* or HTA or HTAs).tw,kw. (452294)

95 biomedical technology assessment/ (22978)

96 (cochrane or health technology assessment or evidence report).jw. (49456)

97 (network adj (MA or MAs)).tw,kw. (18)

98 (NMA or NMAs).tw,kw. (4394)

99 indirect* compar*.tw,kw. (5457)

100 (indirect treatment* adj1 compar*).tw,kw. (666)

101 (mixed treatment* adj1 compar*).tw,kw. (1388)

102 (multiple treatment* adj1 compar*).tw,kw. (368)

103 (multi-treatment* adj1 compar*).tw,kw. (4)

104 simultaneous* compar*.tw,kw. (2191)

105 mixed comparison?.tw,kw. (100)

106 or/90-105 (807237)

107 89 and 106 [BETA LACTAM ABX - ADMINISTRATION - REVIEWS] (2008)

108 exp animal/ or exp animal experimentation/ or exp animal model/ or exp animal experiment/ or nonhuman/ or exp vertebrate/ (49263583)

109 exp human/ or exp human experimentation/ or exp human experiment/ (37877337)

110 108 not 109 (11387925)

111 107 not 110 [ANIMAL-ONLY REMOVED] (2004)

112 editorial.pt. (1066295)

113 letter.pt. not (letter.pt. and randomized controlled trial/) (2050274)

114 111 not (112 or 113) [OPINION PIECES REMOVED] (1983)

115 114 use emczd [EMBASE RECORDS] (1487)

116 Anti-Bacterial Agents/ (462942)

117 exp beta-Lactams/ (129374)

118 beta-Lactam?.ti,ab,kw. (44996)

119 (aztreonam? or az-threonam? or azactam or azthreonam or urobactam).ti,ab,kw. (7136)

120 carbapenem?.ti,ab,kw. (25137)

121 (cefepime or axepim or BMY 28142 or BMY-28142 or cefepim or maxipime or quadrocef).ti,ab,kw. (7908)

122 cephalosporin?.ti,ab,kw. (47896)

123 (imipemide or imipenem? or MK-0787 or MK0787 or N-Formimidoylthienamycin).ti,ab,kw. (23793)

124 (meropenem? or merrem or penem or ronem or SM 7338).ti,ab,kw. (15909)

125 (monobactam? or mono bactam?).ti,ab,kw. (1657)

126 (moxalactam? or mox alactam? or disodium latamoxef or festamoxin or lamoxactam or latamoxef or shiomarin).ti,ab,kw. (3425)

127 penicillin?.ti,ab,kw. (134951)

128 (piperacillin? or pipcil or pipracil or pipril or T-1220 or T1220).ti,ab,kw. (16434)

129 (tazobactam? or taszobactam sodium or YTR 830 or YTR 830H).ti,ab,kw. (11329)

130 thienamycin?.ti,ab,kw. (768)

131 or/116-130 [BETA-LACTAM ABX] (708641)

132 Drug Administration Routes/ (12844)

133 Drug Administration Schedule/ (148272)

134 drug administration*.ti,ab,kw. (119115)

135 exp Administration, Intravenous/ (521446)

136 infusion?.ti,ab,kw. (569830)

137 intravenous*.ti,ab,kw. (781102)

138 (bolus or boluses).ti,ab,kw. (126175)

139 ((continuous* or daily or extend* or intermittent* or longer or "multiple-daily" or "once-daily" or optimal* or optimis* or optimiz* or prolong* or "short-course" or "short-term") adj3 (administer* or administration*)).ti,ab,kw. (110060)

140 ((continuous* or daily or extend* or intermittent* or longer or "multiple-daily" or "once-daily" or optimal* or optimis* or optimiz* or prolong* or "short-course" or "short-term") adj3 (dosage* or dose or doses or dosing or regimen? or infus* or IV or "I.V.")).ti,ab,kw. (327576)

141 (method? adj2 (adminster* or administration*)).ti,ab,kw. (12968)

142 (route? adj2 (adminster* or administration*)).ti,ab,kw. (36609)

143 Anti-Bacterial Agents/ad [administration & dosage] (48909)

144 exp beta-Lactams/ad [administration & dosage] (20798)

145 or/132-144 [DRUG ADMINISTRATION] (2131045)

146 131 and 145 [BETA-LACTAM ABX - ADMINISTRATION] (106689)

147 146 use coch,dare,clhta [COCHRANE DATABASES] (38)

148 56 or 115 or 147 [ALL DATABASES] (3603)

149 limit 148 to yr="1990-current" [Limit not valid in DARE; records were retained] (3574)

150 remove duplicates from 149 (3077) [TOTAL UNIQUE RECORDS]

151 150 use medall [MEDLINE UNIQUE RECORDS] (2006)

152 150 use emczd [EMBASE UNIQUE RECORDS] (1035)

153 150 use coch,dare,clhta [COCHRANE UNIQUE RECORDS] (36)
