# Supplementary material for: Clinical and pharmacokinetic/dynamic outcomes of prolonged infusions of beta-lactam antimicrobials: An overview of systematic reviews
Source: PLoS One. 2021 Jan 22;16(1):e0244966. doi: 10.1371/journal.pone.0244966 (PMC7822342; doi:10.1371/journal.pone.0244966)
Supplement: Attachment — Submitted filename: Response to reviewers 25.10.2020.docx [file pone.0244966.s013.docx]

Clinical and pharmacokinetic/dynamic outcomes of prolonged infusions of beta-lactam antimicrobials: an overview of systematic reviews

Pierre Thabet ^1^, Anchal Joshi, Erika MacDonald ^1^, Brian Hutton ^2,3^, Wei Cheng ^2^, Adrienne Stevens ^4^, Salmaan Kanji ^1,2^

**Response to Reviewers**

Author’s response is in *italics*.

*Thank you for this thorough review of our manuscript and consideration of publication in PLOS-One. Please find below our itemized responses to your comments.*

**Journal Requirements:**

1. Please ensure that your manuscript meets PLOS ONE's style requirements, including those for file naming. The PLOS ONE style templates can be found at

<https://journals.plos.org/plosone/s/file?id=wjVg/PLOSOne_formatting_sample_main_body.pdf> and

<https://journals.plos.org/plosone/s/file?id=ba62/PLOSOne_formatting_sample_title_authors_affiliations.pdf>

*Authors’ Response: done.*

2. It appears there are two cases of broken references, one at the beginning of the results section, and one in the section which begins: "Nine of 14 reviews (21, 22, 26-29, 33, 45, 50) show lower mortality with prolonged infusions...". There are also some comments which I think are left over from drafting your paper ("Also would suggest you define what the ‘N’ and ‘u’ are referring to in the caption. I would also indicate in one sentence what the pattern shown is saying about these reviews to spell out for readers.") in the Findings, Mortality section. Please can you fix or remove these in your revised manuscript.

*Authors’ Response: Thanks for identifying the superfluous text. It has been removed. The broken references have been corrected.*

3. Please amend either the title on the online submission form (via Edit Submission) or the title in the manuscript so that they are identical.

*Authors’ Response: Good catch. We have corrected the title in the manuscript to match the title uploaded.*

4. Please include a caption for figure 1.

*Authors’ Response: the caption is “Results of systematic review identification, screening and inclusion”. The figure title is in bold.*

**Reviewer #1:**

1. Line 61 – 62: agree but please include references to support this.

*Authors’ response: Reference added as per your suggestion.*

2. Line 63: such as drug clearance, metabolism and volume of distribution – felt like an incomplete and a hanging statement? Maybe the authors should add differences after i.e. …volume of distribution differences.

*Authors’ response: We agree that the wording of this sentence could be improved. We have applied your suggestion.*

3. 40 – 50% fT>MIC: is this true for cephalosporins, where 60-70% fT>MIC was associated with better outcomes including in animal models and human studies. Clarifications/amendments are needed.

*Authors’ response: You are right. Attempts to generalize this comment have resulted in inaccuracies. When grouping all beta-lactams together the range should be 40-70%. This range depends not only on the class of beta lactam but also in some cases the organism too. We have attempted to clarify this statement and provide an additional reference as per your suggestion.*

4. 100% fT>MIC: please add better references to support this e.g. McKinnon 2008 etc. References 11 and 12 didn’t identify this target.

*Authors’ Response: Agreed. We have added Peggy McKinnon’s reference as well as a couple others including the French Guideline paper recommending (strong agreement) 100% fT>MIC.*

5. Line 79: intravenous

*Authors’ Response: Corrected, thank you.*

6. Line 79 – 80: minor – I don’t really agree with prolonged infusion changing PK, PK remains the same e.g. Vd and CL doesn’t change with method of administration but PK/PD target attainment does change. I suggest modifying this.

*Authors Response: Agreed. Modified as per your suggestion.*

7. Discussion: “these differences preclude us from broadly generalizing the benefits of prolonged infusions of beta-lactam antimicrobials” – this notion is already known and has been described in many reviews and studies previously i.e. prolonged or continuous infusion won’t benefit all patients but only a sub-group of patients who are severely ill. My question to the authors is what is new and so important with their findings and review of reviews then? This needs to be clarified and described clearly as presently, this study despite using a slightly different methodology is also reporting and describing similar results with existing studies.

*Author’s Response: This is a fair comment. As per comments from another reviewer we have clarified our research question and as per your suggestion have added some text to the discussion with more specific observations. We (and most others) struggle regarding the role of overviews or umbrella reviews. According to the Cochrane handbook there are 4 reasons to conduct an overview: 1)to summarize the evidence for outcomes of different interventions for the same condition, 2)to summarize the evidence of different outcomes for the same intervention 3)to summarize the evidence of the same intervention for different conditions or populations and 4)summarize the evidence of adverse events. We feel that all 4 of these criteria apply to some extent. Unfortunately, due to variability in conduct and reporting, availability of data etc, not all of these goals were achieved. Here are the findings of our overview that we synthesized, and I think are not obvious when reading individual SR/MAs. 1)The populations studied most often in reviews where a mortality/clinical cure benefit are critically ill patients and those with life-threatening infections. 2)The evidence is greater in support of penicillins and carbapenems (particularly pip-tazo and meropenem) rather than cephalosporins. 3)Data from observational studies appear to systematically introduce bias and given the amount of RCT data further analyses should focus on RCTs. 4) we also identify numerous gaps worthy of further research (i.e., prolonged versus continuous infusion, cost-effectiveness, correlation between PK and clinical outcomes and adverse events. We have added some text to the discussion to draw attention to these findings. We have also added text to the introduction clarifying our choice of methods and provided a more specific research question.*

8. Most review combined EI and CI so hard to comment between the two strategies – the authors could comment on PK/PD target attainment between the two?

*Authors’ Response: This was a good thought. We did go back and review the reviews that reported on PD target attainment and none of them separated continuous infusions from extended infusions. Without this we would need to go back to primary studies which would be beyond the scope of our overview (but would be good to specifically look for in a future traditional SR). We added a statement to the discussion to this effect.*

9. Studies form different countries/continents can also be highlighted. E.g. the ones that would benefit more from prolonged infusion are those from countries with less susceptible pathogens vs. Europe and North America.

*Authors’ response: Another good idea. Upon a closer look we were unable to identify any trends to this effect the trials within each review were not grouped for sub-group analysis and every review had studies from multiple countries.*

**Reviewer #2:**

Abstract:
-I think it is helpful in the objective to report the condition(s) being investigated as well as the population of interest, i.e., beta-lactam infusions for which condition(s) and for whom? In this case I think it would be hospitalized patients (adults?) with infections (any infection? sepsis or septic shock?).

*Authors’ response: We agree that additional description improves the clarity of our message. We have incorporated your suggestion.*

Introduction:
-Line 56: Be careful to keep the verb tense consistent (as much as possible) throughout the main text. This sentence now uses past tense - do you mean 'has increased at a staggering pace'?

*Authors’ Response: Agreed. Changed as per your suggestion.*

Methods:
-I suggest justifying why the Jadad guidance was selected as a framework for dealing with discordant systematic reviews, when modern guidance exists that is tailored specifically toward the conduct of overviews of reviews (the Jadad framework references decision making, but being nearly 25 years old is not tailored to overviews of reviews).

*Authors’ Response: We included the tool as part of our methods because it was a peer-reviewed framework that focuses on one particular aspect of the process: interpreting discordance among reviews. Although the framework was developed as a tool to help decision-makers select among available reviews, it has sufficient face validity to use in the context of the overview as it serves the same intent: to distil information to help the decision-maker. When we originally began this project the chapter on overviews from Cochrane was not available and we had to piece together the methods from the existing chapters on systematic reviews that briefly discuss overviews but do not provide detailed instructions for conduct. About halfway through conducting this review the Cochrane chapter was made available to us and we modified our methods to align with the guidance from Cochrane. After some discussion with our methods experts we feel that it is appropriate to remove reference to Jadad and replace it with the Cochrane guidance on overviews as this better aligns to what we actually did. Hopefully this improves the clarity of our methods. We have added this to our deviations from the protocol*

-I suggest justifying why an overview of reviews was the most appropriate methodology for addressing the research question. With just over 60 available trials (as mentioned in the Introduction), why was a systematic review not feasible? Important considerations (as outlined by Cochrane) include whether existing systematic reviews are up-to-date, whether they are sufficiently homogeneous with respect to populations, comparators, and/or outcome measures, and whether existing systematic reviews are of sufficiently low risk of bias (or high methodological quality). The description of available systematic reviews in the Introduction (lines 84-86) is not entirely convincing, so could perhaps be edited to provide a stronger rationale.

*Authors’ Response: At the outset, we were aware of the many reviews that existed and reasoned that it seemed the most intuitive to consider what has already been conducted, given energy that was poured into this topic already. A benefit of overviews as a design type is that it can serve as a knowledge translation vehicle to have collated and distilled reviews across at least one dimension of breadth, in one place.*

*The undertaking of an overview, however, is a complex one. We feel as a team that we explored the necessary characteristic across reviews. Could we have better served the clinical community by having undertaken a review? This seems logical based on the results of the overview, but we would not have known this at the outset. Further, we are aware of sixty studies, but with a proper systematic search may have uncovered many more, for which the totality of a proper synthesis (including presumably a network meta-analysis) would not have been feasible without research funding. That said, the conduct of this overview was no small feat, and we invested more energy than was anticipated.*

*However, considering this reviewer’s feedback, we have expanded on describing why an overview would be undertaken, why we think the topic was suitable for an overview, and further specified our research question, for clarity. Certainly, we hope the benefit of having conducted this overview is to highlight the issues we raised, including the need to increase the credibility of future reviews by using key, best practice methods. This alone, if broadly understood in the research community, will avoid future research waste.*

-I suggest reporting specifically which grey literature sources were searched and the search terms/strategy used.

*Authors’ Response: Added as per your suggestion.*

-If feasible, consider running a search update since the searches are now >1 year old. If this is not feasible due to time and/or resource constraints, you may wish to include this as a limitation.

*Authors’ Response: This was the most stressful search I have ever done! Thankfully we are able to extend our search by a year to June 2020, which keeps the search date within an acceptable period of recency. We did not find another SR that meets our inclusion criteria. We have adjusted the dates and our flow diagram accordingly.*

-It would be helpful to the reader if somewhere within the main text (ideally the introduction or beginning of the methods) you included an explicit, well-defined research question, including the population, intervention(s), comparator(s), outcome(s), length of follow-up, and setting(s) of interest for the overview of reviews. At present, it is not entirely clear.

*Authors’ Response: As mentioned before, this has been added to the end of the introduction as per your suggestion.*

-I suggest providing a more detailed description of the methods used to deal with missing, flawed, or discordant risk of bias assessments for the same studies across systematic reviews. Similarly, it would be helpful to report in more detail how you dealt with discrepant data from primary studies within the included systematic reviews during data extraction.

*Authors’ Response: In our methods we stated that we would extract study level ROB assessments as they were reported (when they were reported) for each review, preferably at the outcome level but also at the review level. Due to resource limitations we did not conduct our own ROB assessments at the study level, but extracting as-is from reviews, to the best of our ability despite potential variability across reviews and is outlined as an acceptable approach in the recently updated Cochrane overviews guidance. We describe the variability in the results section and even provide a sample table of 10 trials describing how in some cases 5 different methods of ROB assessment were used for the same study across 5 different reviews. If we had done our own, we could have commented on discrepancies between our interpretation and those of the authors but with the sheer volume of studies this was beyond what we could do with the resources we had. At the time we decided that the added value was not worth the investment of time on our part given the challenges of other overview authors in interpreting this data.*

-As AMSTAR-2 and ROBIS include many overlapping items (despite aiming to measure different constructs, empirical evidence shows that the overall appraisals tend to be correlated), it would be helpful to justify why both were used instead of one or the other (which would have been sufficient).

*Authors’ response: To our knowledge there is no best practice guidance to choose one tool over another; further, the authors of the Cochrane handbook chapter indicate that they cannot currently recommend one tool over another due to a lack of empirical evidence. We agree with you that the overlap between tools introduces elements of redundancy but as we understand it, the intent of these tools are different. Therefore, we included both in our overview, and certainly our assessments can contribute data to the evidence base around this question of choice. For these reasons, rather than attempt to justify the choice of one over the other we have elected to report both based on pragmatic considerations and to appease all readers given the equipoise on this issue.*

-For AMSTAR 2, it would be helpful to define which domains you considered to be "critical" in the context of this overview of reviews.

*-Authors’ response: As per your suggestion we have identified 7 domains that we deem critical to this review and have added them to the methods.*

Results:
-I suggest adopting consistent terminology throughout the main text to avoid confusion. For example, in line 150 you note that 21 studies were included when in fact (I believe?) these were unique systematic reviews. It will be important throughout the text to differentiate between the systematic reviews, and the primary studies included in them.

*Authors’ response: Thank you for noticing this. With such a technically oriented manuscript it is imperative that we use the right words! I only noticed one other instance where we mixed up review and study.*

-Table 1: Just a detail, but technically AMSTAR 2 is used to appraise your confidence in the results of the systematic review (not 'quality' per se). I would suggest revising the title of the final column of the table to reflect this.

*Authors’ response: We have changed to title to “Critical appraisal and risk of bias” as per your suggestion.*

-Table 1: Since 'quality' or 'confidence' in review findings should (in theory?) be inversely related to risk of bias, you will need to explain how many of the systematic reviews appear to be of low or critically low quality, while also being at low risk of bias. Although I can appreciate that these are distinct constructs, as mentioned previously, the two tools are relatively similar in the concepts measured, so this will be important to justify. Also, how should readers interpret this discordance?

*Authors’ response: Thank you for inquiring. It appears that most discrepant judgements between the two tools were related to items unique to each tool. We have provided all the AMSTAR-2 and ROBIS assessments for the reader and have added a statement to the results regarding the discordance.*

-Table 1: I would suggest reviewing the contents of the table for accuracy, especially in areas where "unspecified" has been used. For example, a cursory review of the systematic review by Roberts (2009) shows that the population of interest was hospitalized adults, while the table indicates "unspecified population".

*Authors’ response: Thank you for this attention to these details. It is correct that the studied population in the review by Roberts (2009) is hospitalized adults with acute infection – and not simply “unspecified”. All relevant tables have been modified. Further clarity was also provided with regards to the study population for the systematic review by Garcia (2012). The remaining seven systematic reviews with unspecified populations have been verified. No further qualifiers on included study population are provided. As such the qualifier “unspecified” has been retained for these seven systematic reviews.*

-Under the interventions studied, it would be helpful to note, rather than simply "studied", which were the comparisons of interest (since these were all investigating comparative effectiveness?).

*Authors’ response: Text in this section has been added to reflect your suggestion.*

-Can you provide a more detailed description of overlap (i.e., for which outcome was overlap 5% vs. 78%)? This will be very important for the readers to be able to interpret the credibility of the results and conclusions.

*Authors’ response: A more detailed description of overlap is provided for each outcome as a figure and in the narrative.*

-For review quality and risk of bias, please note that GRADE and the Cochrane RoB tool are measuring two separate constructs (GRADE measures outcome-level certainty of evidence, whereas the RoB tool measures study-level risk of bias). I suggest discussing these separately.

*Authors’ response: GRADE was only used by one review. As per your suggestion we have tried to clearly separate these points when they are discussed in the same section.*

-Table 2 is not useful and should likely be deleted. Preferably, the information for all reviews would be included in an Appendix. Further, more work should be done to deal with the discordant assessments, rather than simply writing them off as 'discordant' (which is to be expected).

*Authors’ response: We are happy to delegate this table to an electronic appendix but would rather not delete it entirely as it provides a tangible example to readers of the variability that was observed. As mentioned before, a proper analysis of discordance would require us to determine our own risk of bias at the study level which we did not do (as mentioned previously). We have listed this as a limitation to our overview. We feel that this table describes why we were not able to synthesize this data and augments the text.*

-Table 3 is not adding anything to what has already been described in Table 1, so I would suggest deleting it.

*Authors’ response: We have moved this table to the supplementary appendix.*

-It is not very helpful to provide ORs and RRs without 95% CIs. I would suggest revising to include these.

*Authors’ response: Good point. We have added the 95% CI for those that were missing.*

-Many of the reported results seem to focus on the high variability in the populations, interventions, outcomes, and their definitions, suggesting that perhaps an overview of reviews was not the ideal approach? You may wish to investigate this more carefully. Seems this was a severe limitation precluding strong conclusions.

*Authors’ response: This is a fair assessment, but I would argue that hindsight is 20/20. As we’ve discussed above, we have tried to justify our choice of methods in the introduction. While we anticipated some variability in the research questions behind each review we didn’t really know how much to expect until actually going through the exercise. Despite the fact that we were unable to make strong conclusions I think our methods were sound and this lack of confidence is an important finding on its own in addition to others that we’ve made. While we acknowledge this as a limitation, we strongly feel that this overview makes important observations about the quality and direction of the evidence, identifies gaps in the evidence and makes strong suggestions for moving forward in this field of research.*

-Again, Table 4 is not adding anything to what was already described in Table 1, so I would suggest deleting it.

*Authors response: As for table 3 we have moved this table to the supplementary appendix.*

-It would be helpful instead to present tables summarizing the findings (e.g., pooled estimates of effects) across the various systematic reviews.

*Authors’ response: The tables and figures are meant to complement each other. It is our opinion that the outcome data from each review is better presented in the forrest plots. Regarding pooling, it was never our intention to meta-analyze data between studies or reviews if that is what you mean. I know some overviews will conduct meta-analyses of primary studies found in these reviews but that was beyond the scope of what our resources would allow. Furthermore, we would have needed in depth understanding of the studies across reviews to determine whether pooling across them (and avoiding double counting) would have been appropriate to avoid violating the assumption of transitivity.*

-Figure 1 - I suggest here replacing "studies" with "systematic reviews".

*Authors’ response: Another good catch. We’ve changed it as per your suggestion.*
